# Key roles of GAPDH, Hsp90, and NO in heme trafficking^☆^

**DOI:** 10.1016/j.jinorgbio.2025.113066

**Published:** 2025-09-08

**Authors:** Dennis J. Stuehr, Pranjal Biswas, Yue Dai, Dhanya Thamaraparambil Jayaram, Priya Das Sinha, Elizabeth A. Sweeny, Arnab Ghosh

**Affiliations:** a Department of Inflammation and Immunity, Cleveland Clinic Lerner College of Medicine of Case Western Reserve University School of Medicine, Cleveland 44195, OH, USA; b Department of Biochemistry, Medical College of Wisconsin, 53226, Milwaukee, WI, USA

**Keywords:** Mitochondria, Chaperone, Hemeprotein, Homeostasis, Catalysis, Nitrosation, Nitric oxide

## Abstract

Intracellular trafficking of mitochondrial heme to create functional heme proteins presents a fundamental challenge in animal cells. This article provides some background on heme allocation, discusses some of the concepts, and then reviews research from the last two decades that has uncovered unexpected and important roles for glyceraldehyde 3-phosphate dehydrogenase (GAPDH), heat shock protein 90 (Hsp90), and nitric oxide (NO) in enabling and regulating cell heme allocations to hemeproteins that mature and function outside of the mitochondria. A model for how hemeprotein heme contents and functions in cells can be regulated through the coordinate participation of GAPDH, Hsp90, and NO is presented.

## 1. Introduction

### 1.1. Heme allocation in mammalian cells- what is possible?

Diverse proteins rely on Fe-protoporphyrin IX (heme) to perform functions in electron transfer, small molecule transport, catalysis, and signal detection and propagation [1]. In eukaryotes the mitochondria perform the initial and final steps of heme biosynthesis and typically are the sole source of heme in cells. As discussed by Gallio et al. [2], intracellular distribution of mitochondrial heme is influenced by an overarching thermodynamic driving force because most hemeproteins (cytochromes, globins, catalase, etc.) have relatively high heme binding affinities. Thus, prevailing thought has mitochondrial heme flowing through a pool of exchangeable heme in cells that is available for further allocation and deliveries, ultimately leading to the heme becoming tightly sequestered within final target proteins and effectively no longer in equilibrium with the exchangeable heme pool unless the proteins become degraded. Within this framework cells could achieve specificity in trafficking of mitochondrial heme to proteins outside this organelle by minimizing the amount of free heme that exists and then regulating the behaviors and interactions of proteins or other macromolecules that participate in heme handoffs. This includes imposing spatial constraints on heme distribution (organelles), coupling heme transfers to changes in the heme iron redox state or in its ligation state, having heme-driven structural changes in the macromolecules alter their heme affinities or interactions, and by regulating the kinetic barriers of these processes [2–5]. Thus, the situation would shift from random heme distribution as determined by the thermodynamic gradient to a distribution process where certain pathways become timelier and more probable than others. It is also important to note that mitochondrial heme distribution in living cells never reaches a true equilibrium because cells are constantly generating, destroying, recycling, and exporting heme according to several dynamic and inter-regulated processes [6–12]. As will be described in this review, it now appears that these circumstances do exist and have allowed a small number of individual macromolecules to take on primary roles in the intracellular allocation of mitochondrial heme.

### 1.2. Many hemeproteins naturally reside in their heme-deficient form in cells and tissues

That cells naturally accumulate hemeproteins in their heme-deficient state has not been widely appreciated. The prevailing view has mitochondrially-provided heme ultimately saturating cellular hemeproteins that possess canonical “high affinity” heme binding sites. It follows that such hemeproteins would primarily exist with a full complement of heme except for transient periods when they are being synthesized or undergoing degradation. An example that fits this paradigm is for the hemoglobin-α and -β subunits (Hb-α and -β) expressed in erythrocytes, which were found to be ≥95 % heme saturated [13]. However, this circumstance may be exceptional and limited to erythrocytes. The globins Hb-α, Hb-β, and myoglobin [14] are widely expressed in non-erythroid cells and tissues and in such cases exist less than 50 % heme-saturated [15,16]. Perhaps the oldest example of this behavior is the hemeprotein L-tryptophan dioxygenase (TDO), which was found to exist only 30–40 % heme-saturated in the livers of healthy rats over 70 years ago [17]. Since then, heme saturation levels of less than 50 % have been consistently documented for TDO and also for the related Trp-metabolizing enzyme indoleamine 2,3-dioxygenase-1 (IDO1) in different cell types [18,19]. Results from the literature indicate that hemeproteins including cytochrome P450’s (CYPs) [20–22], soluble guanylyl cyclase beta subunit (sGC-β) [23–25], NADPH oxidase 5 (NOX5) [26], and various peroxidases [27] all exist largely or even predominantly in their heme-deficient forms when expressed in mammalian cells or in certain tissues. For the CYPs, their heme saturation levels appear to vary depending on the animal species and the organ location. For example, the heme saturation levels of CYPs expressed in the liver, kidney, testis, and aorta of rabbits (as assessed by heme-dependent activity measures) ranged in descending order from 90 to 3 % heme-saturated, respectively, while in the same organs from rats the range was 100 to 22 % heme-saturated [21]. In samples of human fetal adrenal, liver, and brain tissues, CYP heme saturation levels were 8, 36, and 42 %, respectively [28]. Besides shifting the dominant paradigm, this circumstance of partial heme saturation presents an additional way for cells to regulate their hemeprotein activities, by changing the heme saturation level of their existing hemeproteins. Notably, this mechanism was proposed to regulate CYP activities by Juchau and colleagues at least 45 years ago [28]. Indeed, as discussed in later sections, cells do regulate the heme levels of their hemeproteins in a dynamic way in response to environmental signals and may utilize this strategy to regulate the activities and biological functions of their hemeproteins in various biological settings.

### 1.3. Multiple systems exist to regulate the level of heme in cells

Numerous systems govern the intracellular heme level, including feedback regulation of heme biosynthesis via control of δ-amino-levulinate synthase (ALAS) activity [29], control of heme export out of the mitochondria [30] or out of the cell [31], and heme degradation by the heme oxygenase 1 and 2 enzymes (HO-1 and HO-2) [14]. Cells dynamically respond to changes in their heme level by changing their ALAS activity to diminish or increase their mitochondrial heme biosynthesis, by induction of HO-1 expression to hasten heme degradation, and by expression changes in a wide variety of other proteins [32]. These control mechanisms can be bypassed or manipulated in several ways. The cell heme level can be artificially increased by providing them with hemin and the heme precursor molecule δ-aminolevulinic acid (δ-ALA) which is generated downstream in the biosynthetic pathway from ALAS and thus supports an unregulated heme production by the mitochondria. Under these circumstances the heme saturation levels of some previously-mentioned hemeproteins expressed in cells (globins, sGC-β, TDO, IDO1, peroxidases, NOX5) all increase considerably within 2 h [15,16,19,26,27,33]. Ways to down-regulate the cell heme level include the overexpression of HO-1 [34], reducing the expression level of mitochondrial or endoplasmic reticulum heme exporters [30,35], or increasing the expression level of cell membrane heme exporters [31]. Given such multiple opportunities for regulation, it is fascinating that cells keep their exchangeable heme levels too low to saturate their hemeproteins.

### 1.4. Heme allocation involves its delivery and insertion into apo-hemeproteins

Post-translational heme insertion is a fundamental step in hemeprotein maturation to functional form. Crystal structures of hemeproteins often show their heme is sequestered within the protein with restricted solvent access. Although this arrangement helps explain why such hemeproteins display relatively high heme binding affinities due to their having slow rates of heme dissociation, it does not explain how the heme got into the apo-protein in the first place, except to indicate that the heme could not have entered if the apo-protein was in the conformation as exhibited by the holo-protein. In cases where an analogous structure for the apo-hemeprotein is available, it often indicates it is relatively less-structured and more dynamic, consistent with it having greater susceptibility toward proteolysis or having an increased level of Trp fluorescence emission. In many cases, heme can be introduced into purified apo-hemeproteins by simply incubating them with hemin, but this does not mimic the heme insertion process that occurs inside cells, where the incoming heme is likely to be bound to and transferred by a macromolecular carrier. These considerations all suggest it would be useful for cells to employ chaperone systems to help stabilize the apo-hemeproteins in a receiving conformation and to enable the heme deliveries and insertions, and this appears to be exactly what nature has done.

### 1.5. Proteins involved in intracellular heme transport

Because free heme is redox active and displays highly promiscuous binding properties, its intracellular transport has long been expected to involve macromolecular carriers [1,2]. For a macromolecule to function as a heme chaperone it must be shown capable of binding and accumulating mitochondrially generated heme in cells, and to have its heme binding property linked to downstream heme deliveries. Beyond this, finding that the candidate heme chaperone associates with apo-hemeprotein recipients provides additional justification and some possible mechanistic information. Of all the proteins or other macromolecules that have been considered over the years as possible intracellular heme chaperones [7], to date only one, glyceraldehyde 3-phosphate dehydrogenase (GAPDH), displays all three of the attributes noted above.

### 1.6. GAPDH functions as an intracellular heme chaperone

**Fig. 1.**
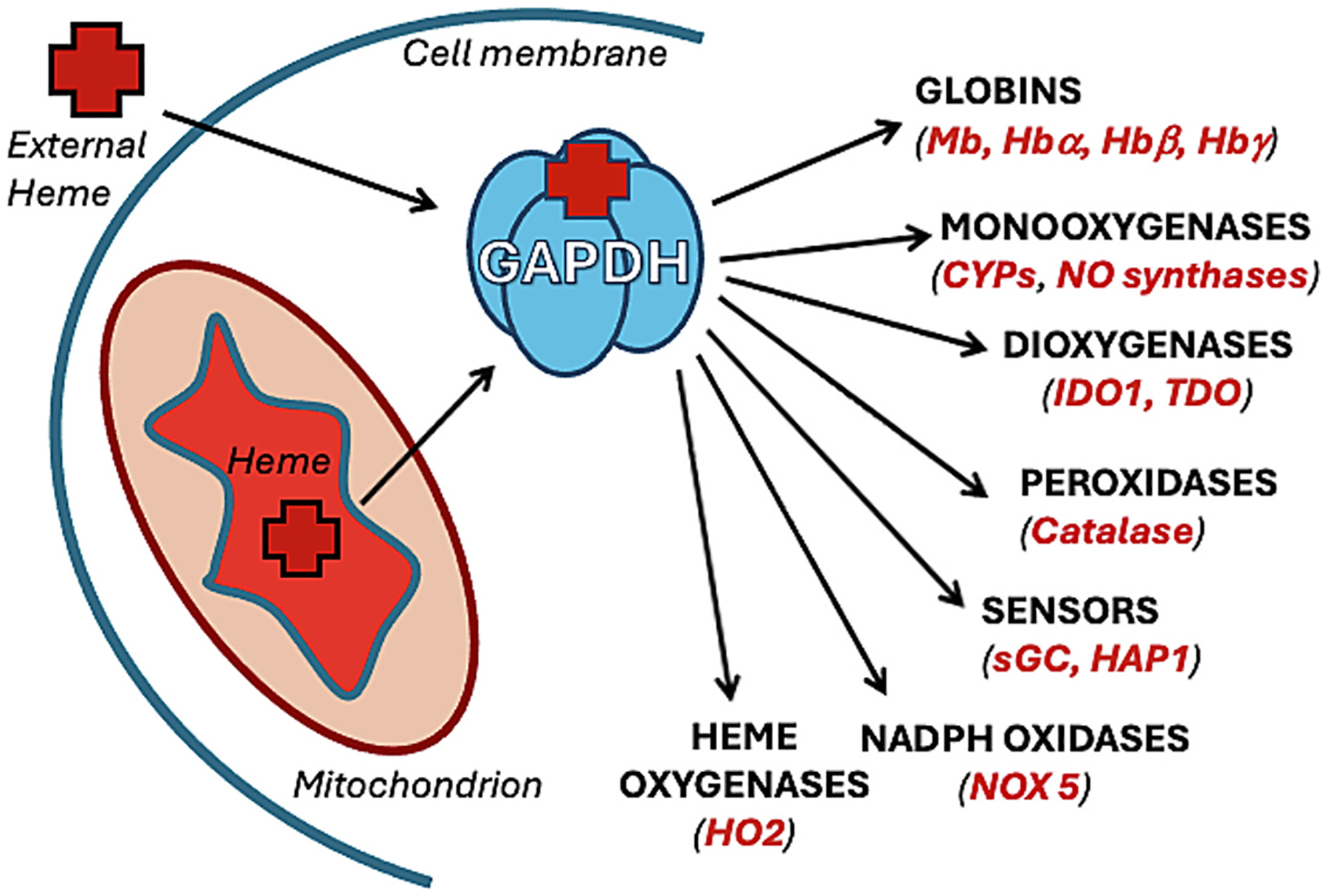
The GAPDH-heme complex plays a broad role in cell heme trafficking. Heme that is provided externally or by cell mitochondrial biosynthesis binds to GAPDH in the cytosol to form a GAPDH-heme complex that traffics heme to many different hemeproteins within the cell. Hemeproteins whose heme deliveries are known to be GAPDH dependent are shown.

GAPDH is a soluble protein that in mammals exists as a homo-tetramer expressed at relatively high levels, for example in rats ranging from 0.5 to 10 % of the total cellular protein depending on the tissue source [36]. At first glance, GAPDH participating in cell heme allocation seems odd because its hallmark function is to act as a dehydrogenase in glycolysis, but GAPDH is already known to have several alternative or “moonlighting” functions [37,38]. The connection between GAPDH and intracellular heme transport first arose from investigations of how NO inhibits cellular heme allocation to inducible NO synthase (iNOS) [39] and to Hb, Mb, CYPs, and catalase [40]. GAPDH was distinguished as a cytosolic protein who’s binding to hemin-agarose resin became inhibited if cells had been exposed to relatively high NO concentrations. This uncovered its potential involvement in heme binding and in how NO had inhibited the heme deliveries [41]. Another study [42] showed that GAPDH bound heme through a mono- or bis-His ligation and its heme binding displayed moderate affinity (150 nM Kd), consistent with GAPDH possibly functioning to traffic intracellular heme based on reported estimates of the “free” heme concentration in cells [43,44]. A genetic screening approach found that heme delivery to the nuclear transcription factor HAP1 in yeast depended on the expression of one of three GAPDH genes [43]. GAPDH was subsequently shown to bind and accumulate heme in mammalian cells, and through an approach involving knockdown of cell GAPDH expression and the use of a GAPDH variant that has defective heme binding (H53A GAPDH) but otherwise possesses normal glycolytic dehydrogenase activity, the heme binding property of GAPDH was linked to intracellular heme deliveries to iNOS in mammalian cells and to HAP1 in yeast [45]. Using the same approach, GAPDH and its heme-binding ability has been shown essential for mammalian cell heme allocations to a wide range of hemeproteins including sGC-β [33], IDO1 and TDO [19], Mb and Hb-α, − β, and -γ [46], HO-2 [47], CYP 2D6 and 3A4 [22], and catalase (Sinha Das and Stuehr, unpublished) (Fig. 1). Thus, evidence to date strongly implicates GAPDH acting as a heme chaperone in eukaryotes and identifies the GAPDH-heme complex as a common intermediate in the pathway for mitochondrial heme distribution to a wide variety of apo-hemeprotein targets in cells.

Recently, a fluorescent GAPDH reporter protein (TC-GAPDH) was developed to study GAPDH heme trafficking. Upon labeling TC-GAPDH with the FlAsH reagent [48], its heme binding can be detected as a proportional quenching of its fluorescence emission [49]. Using TC-GAPDH, it was confirmed that the tetramer contains two heme binding sites of equivalent affinity (Kd = 68 ± 20 nM), consistent with the proposed structural model of the heme-bound GAPDH tetramer [45,49]. When TC-GAPDH was expressed in a human cell line it allowed an estimate of how much heme is bound in GAPDH in cells under normal cell culture conditions (approximately 0.2 heme/GAPDH tetramer, or 1 heme bound per 5 GAPDH tetramers), and revealed that stimulating cell mitochondrial heme biosynthesis caused a gradual 3-fold increase in the level of GAPDH-bound heme to reach 0.6 heme/tetramer [49]. This change was accompanied by a similar increase in the total heme content of the cells and by an increase in the heme contents of two GAPDH client hemeproteins expressed in the same cells [49]. Overall, these findings suggest that cells maintain a pool of heme-containing GAPDH whose heme level can change in league with an increase in cell mitochondrial heme biosynthesis, and this change in turn is reflected in changes to the heme levels in at least two GAPDH client hemeproteins. Thus, the GAPDH-heme complex is likely a pool of exchangeable heme in cells that acts as an intermediate for the allocation of mitochondrially-generated heme through the cytosol to apo-hemeprotein targets in cells (Fig. 1).

### 1.7. How might GAPDH participate in heme trafficking?

The role that GAPDH plays in cell bioenergetics does not appear to be linked to its role in heme trafficking, because neither its heme binding nor the targeted ablation of its heme binding ability (H53A GAPDH) alters GAPDH dehydrogenase activity or causes changes in the cell energy level [41,45]. On one hand, GAPDH could simply act as a passive heme depot that provides a pool of exchangeable heme in cells, where middlemen proteins provide it with mitochondrial heme and other middlemen proteins take its heme away to make the final downstream deliveries. On the other hand, GAPDH could play a more active role by directly receiving heme from the mitochondria [50] and then directly delivering it to its various client hemeproteins [51], with no involvement of middlemen proteins. It could also act between the two extremes, in which middlemen proteins either provide it with mitochondrial heme or deliver its heme to clients. All these possible models would require that GAPDH display considerable promiscuity in its binding with protein partners, whether they be middlemen proteins and/or its various apo-hemeprotein clients. Notably, GAPDH is already known to display promiscuous binding as demonstrated by it making functionally consequential interactions with dozens of different proteins and RNA partners [52].

### 1.8. Are GAPDH-dependent heme deliveries to clients direct or indirect?

Allocation of GAPDH-bound heme to its apo-hemeprotein clients can be considered a “downstream” step in the process of heme trafficking, because it results in heme incorporation into clients for their consequent maturation to functional form. Studies using purified versions of GAPDH and its client apo-hemeproteins show that they interact and form complexes that display single μM affinities, and that GAPDH preferentially binds to the heme-free versus heme replete client forms both when they are studied in their pure form or when they are expressed in mammalian cells [19,33,51]. Indeed, in a reconstitution system using purified proteins, heme transfer from a pre-made GAPDH-heme complex to the client hemeprotein apo-sGC-β was facilitated by their direct contact [33,53], and GAPDH-dependent heme delivery to client apo-IDO1 in mammalian cells was recently shown to require their direct complex formation that is mediated through a specific charge pairing interaction that forms across their protein-protein interface [51]. Thus, studies to date suggest that GAPDH-dependent heme deliveries to at least some apo-hemeproteins require that GAPDH form a direct complex with the client, thus discounting delivery mechanisms involving middlemen proteins. Additional studies to determine what other client’s heme allocations proceed through direct GAPDH complex formation, and the molecular details of their interactions, would further our understanding of this fundamental downstream step in heme trafficking.

### 1.9. How does GAPDH obtain mitochondrially generated heme?

Transfer of mitochondrially-generated heme to GAPDH can be considered an “upstream” step in the heme trafficking process because it forms the GAPDH-heme complex that enables “downstream” heme deliveries to numerous hemeprotein clients. As noted above, in principle GAPDH could procure heme either directly or indirectly from the mitochondria. A recent study [50] has revealed that mitochondrial heme transfer to GAPDH occurs via the mitochondrial membrane transporter protein Feline leukemia virus subgroup C receptor-b (FLVCR1b), which in cells is targeted to the mitochondria [30] and is present in both the inner and outer mitochondrial membranes [30]. Structurally, FLVCR1b is a truncated version of FLVCR1a, which itself is a member of a large family of integral membrane small molecule transporters [54]. Originally, targeted knockdown of FLVCR1b expression in cells had been found to increase the heme level in cell mitochondria and to block the distribution of mitochondrial heme for Hb production during erythropoiesis [55]. Subsequently, the targeted knockdown of FLVCR1b expression was shown to prevent mitochondrial heme from reaching TC-GAPDH in cells and to also block heme deliveries to two GAPDH hemeprotein clients that were expressed in the cells [50]. Besides confirming that the two client apo-hemeproteins have GAPDH-dependent heme deliveries, this study revealed that FLVCR1b is likely the sole conduit for mitochondrial heme to reach GAPDH in cells. In the same study, GAPDH was found to associate with FLVCR1b on the mitochondrial surface to an extent that depended on the level of FLVCR1b expression, and their association was found to be temporally dynamic, increasing when cell mitochondrial heme biosynthesis was stimulated and then decreasing as the heme loaded into GAPDH. These changes in FLVCR1b-GAPDH association and heme uptake by the GAPDH were temporally linked in turn to heme becoming incorporated into two client apo-hemeproteins expressed in the cells. The same study identified a protein named Transport and golgi organization 2 homolog (TANGO2) to also be required for mitochondrial heme transfer into GAPDH and for the downstream heme deliveries. TANGO2 was found to interact with FLVCR1b, suggesting how it may possibly facilitate mitochondrial heme export by FLVCR1b to GAPDH [50]. Thus, GAPDH appears to obtain mitochondrial heme via the FLVCR1b heme exporter through a mechanism that involves their association on the mitochondrial surface. Besides discounting a role for middlemen proteins in this upstream heme transfer to GAPDH, the findings explained why knockdown of cell FLVCR1b or TANGO2 expression had been previously found to prevent heme from reaching Mb or Hb [55]. Both of these globins depend on GAPDH for their heme allocation [46]. Further investigations of FLVCR1b heme export to GAPDH are awaited with interest.

### 1.10. The cell chaperone Hsp90 drives heme insertion into many apo-hemeproteins

**Fig. 2.**
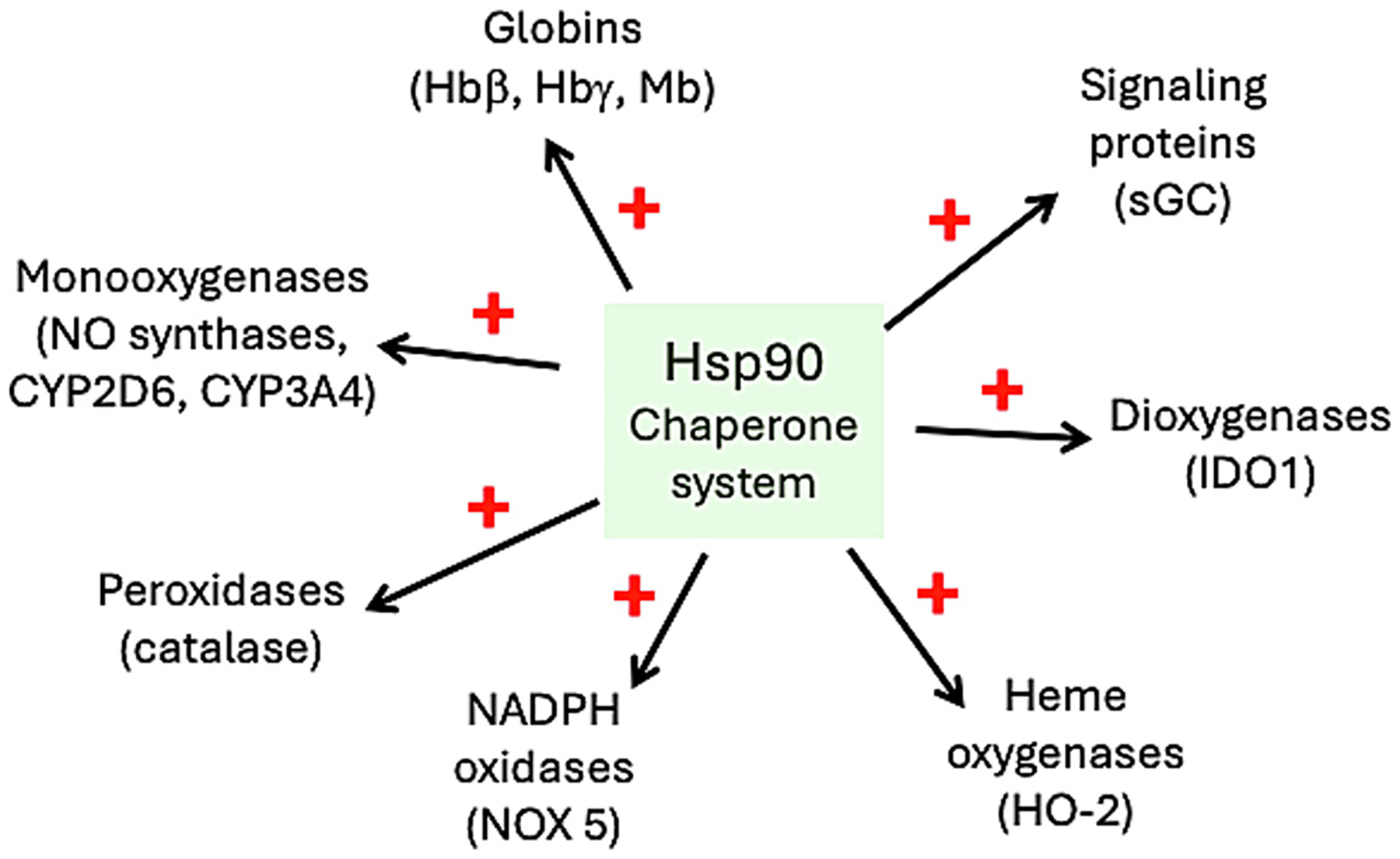
Chaperone Hsp90 enables heme insertion into a broad range of hemeproteins. In mammals, the heme-free forms of hemeproteins are typically in complex with the cell chaperone Hsp90, which is needed to drive their heme insertions. Hemeproteins whose heme insertions are known to be Hsp90-dependent are shown.

The final step in intracellular heme trafficking is the insertion of the heme into the apo-hemeprotein targets. In mammals, a ubiquitous cytosolic chaperone protein named heat shock protein 90 (Hsp90) has been discovered to play a broad role in driving these heme insertions. In general, Hsp90 and its related co-chaperone proteins are utilized in eukaryotes to assist in final folding of many protein clients [56] and to assist in insertion of small molecules into proteins, as demonstrated for steroid binding into its receptor [57]. The first evidence that Hsp90 or related co-chaperones might be involved in cellular heme trafficking came from studies showing that it helped regulate functions of heme-dependent signaling kinases and transcription factors [58,59]. Subsequent studies showed that Hsp90 associated with endothelial NOS and sGC hemeproteins in cells and this correlated with their enzyme activities, which was presumed due to Hsp90 binding and affecting their mature heme-containing forms [60,61]. However, it now seems clear that Hsp90 predominantly influences the functions of these two and a host of other hemeproteins through it binding to their immature heme-free forms to drive the heme insertions required for their functional maturation [62–64]. Along with the two hemeproteins noted above, Hsp90 has been shown to interact with the heme-free forms of myoglobin, Hb-β, Hb-γ, IDO1, and CYP2D6 and CYP3A4 in cells [15,16,19,22] and to be essential for driving their heme insertions (Fig. 2). Indeed, among all the hemeproteins studied to date only one (TDO) has been found to have an Hsp90-independent heme insertion in cells [19]. Thus, Hsp90 has emerged to play an important role in heme trafficking by it functioning to drive heme insertions into many apo-hemeproteins, thereby enabling their maturation to functional forms.

### 1.11. Mechanism of Hsp90 action

Hsp90 has a high level of binding promiscuity as is demonstrated by it binding to well over one hundred protein clients [56,65] including the apo-hemeproteins noted above. Hsp90 displays single μM affinity toward binding with its apo-hemeprotein clients, binds more strongly when the clients are in their heme-free states, and dissociates from the clients after their heme insertions have taken place [15,16,19,24,25]. The functions of Hsp90 typically require its ATPase activity [56]. Although its ATPase activity is not required for Hsp90 to bind with its apo-hemeprotein clients it is needed to drive their heme insertions in cells [19,24,63,64]. ATP hydrolysis causes the structural rearrangements and cycling in Hsp90 that enable its chaperone functions [56,66] and this is likely how ATP hydrolysis drives the Hsp90-assisted heme insertions. It is thought that Hsp90 binding, perhaps along with its ATP-ase activity, may help to hold the apo-hemeproteins in conformations that are more amenable for heme insertion [62,67]. So far, the molecular aspects of Hsp90 interaction and influence have only been studied for two hemeprotein clients: neuronal NOS [68] and apo-sGC-β [69–71]. Regarding sGC-β, studies identified regions that enable its Hsp90 complex formation [69]. This led to a model structure of the Hsp90-apo-sGC-β complex in which structural elements located in the middle domain of Hsp90 interact with structural elements in two regions of the per-arntsim [72] domain of apo-sGCβ [69]. Targeted mutagenesis within these regions of sGC-β designed to diminish its Hsp90 interaction did so as predicted by the structural model [69], supporting its accuracy. Spectroscopic data and the model indicated that Hsp90 likely forces the heme binding domain of apo-sGC-β to exist in a more solvent exposed state so it can better accept heme incoming from a delivery entity like GAPDH [67]. Further work with sGC-β variants demonstrated that a Hsp90-aposGC-β complex that is similar to the model must form in mammalian cells in order for apo-sGC-β heme insertion to occur [70]. Reconstitution studies using purified forms of GAPDH, Hsp90, and apo-sGC-β or aponNOS client proteins were recently performed to shed light on how Hsp90 impacts heme transfer from a pre-formed GAPDH-heme complex [53,68]. Results showed that the GAPDH-bound heme directly transferred into apo-sGC-β or apo-nNOS. When Hsp90 was bound to the aposGC-β it slowed the rate of its heme uptake from GAPDH, but when the ATP-ase activity of Hsp90 was engaged, it sped its rate of heme incorporation by 2 or 3 fold [53]. This revealed that Hsp90-based conformational changes in the client apo-sGC-β protein could either negatively or positively regulate its rate of heme incorporation from GAPDH.

Given that Hsp90 dissociates from its hemeprotein clients after they have incorporated heme, a conformational change in the client protein that follows its heme incorporation is likely what diminishes its Hsp90 binding affinity. In principle, this heme incorporation effect on Hsp90 binding provides cells with a way to control when their hemeproteins can form complexes with their partner proteins or subunits, as if often required to achieve biological function. For example, Hsp90 binding to the heme-free subunits of Hb-β, Hb-γ, NOS, or sGC-β ensures that they cannot bind to their respective partner subunits until after the Hsp90-bound apo-hemeprotein incorporates heme. In this way, Hsp90 can direct the maturations of functional Hb-αβ or Hb-αγ tetramers, sGC-αβ heterodimer, and NOS homodimers. This has been most clearly demonstrated in Hsp90 directing functional sGC heterodimer formation [70]. Thus, Hsp90 appears to play two distinct roles in hemeprotein biology: It drives their heme insertions and also ensures that the apo-hemeproteins do not interact with their protein partners until after they have received heme.

**Fig. 3.**
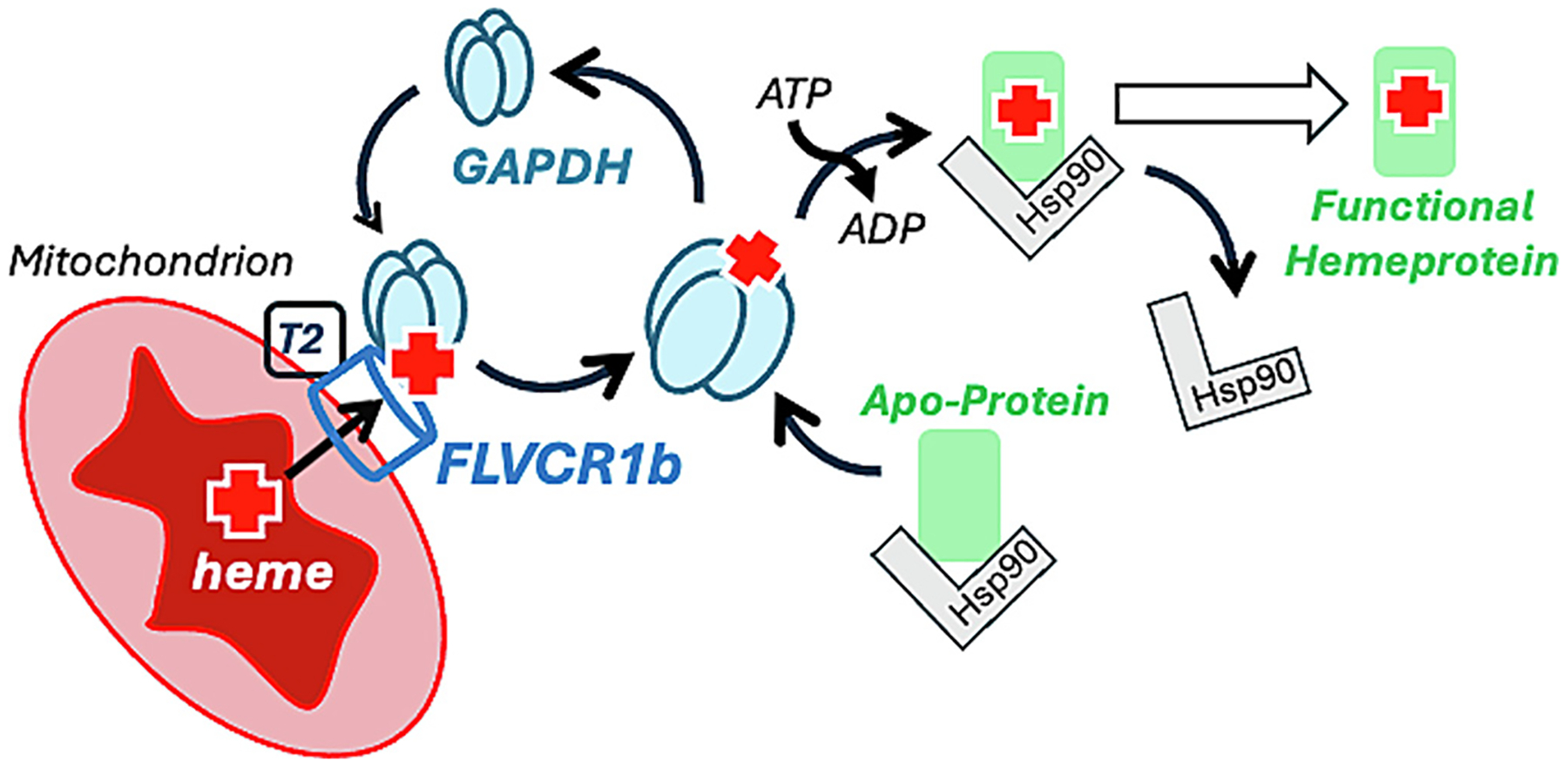
Model for mitochondrial heme allocation within mammalian cells. and NO in regulating the level of a functional hemeprotein (sGC) in cells. The evidence suggests that mitochondrial heme allocation relies on a relatively simple pathway involving four principal proteins. Two mitochondria-associated proteins (the FLVCR1b integral membrane transporter and TANGO2, shown as T2) enable mitochondrial heme to be exported to a third protein (cytosolic GAPDH) that can broadly distribute heme in cells. A fourth protein (Hsp90) is bound to the apo-hemeproteins and enables their uptake of GAPDH-derived heme through an ATP-driven process, thus forming functional hemeproteins.

A model for mitochondrial heme allocation in mammalian cells. As illustrated in Fig. 3, findings to date suggest that heme allocation in eukaryotic cells may rely on a relatively simple pathway that involves four principal proteins: Two mitochondria-associated proteins (FLVCR1b and TANGO2) that enable mitochondrial heme export to a third protein (cytosolic GAPDH) that can broadly distribute heme in cells, and a fourth protein (Hsp90) that enables GAPDH-derived heme to be inserted into the heme protein clients. This pathway as outlined may improve our general understanding of heme biology and may guide further investigation of the mechanisms that control heme allocation in mammals.

### 1.12. The heme levels in hemeproteins can change in response to environmental signals

Several hemeproteins that perform signaling functions (for example, heme-regulated kinase, various heme regulated transcription factors) have evolved to respond to changes in the cell heme level and thus must naturally exist in variable heme-deficient states [73]. As noted previously, many if not most other hemeproteins that exist in non-erythroid cells and tissues are a mix of their heme-deficient and heme-replete states. Beyond stimuli that increase cell heme biosynthesis or change cell HO-1 expression levels, other biological signals can change the heme saturation level of hemeproteins. In the case of liver TDO, it exists naturally at about 40 % heme saturated, and provision of its substrate L-Trp to live animals or to liver homogenates significantly increased its heme saturation level to 90–100 % [74]. Inflammation often alters hemeprotein activities and this effect has often been ascribed to changes in hemeprotein expression levels, but it could also arise due to changes in their heme contents [75]. Inflammation in mammals is often accompanied by increased NO biosynthesis and as discussed below, NO can and does have significant impact on the heme levels of hemeproteins to consequently alter their activities and functions.

### 1.13. NO can impact hemeproteins by multiple mechanisms

The discovery of NO biosynthesis in mammals [76] created interest in how such endogenous NO might impact metalloprotein functions in biology. NO can impact hemeproteins in at least three ways: It can directly ligate to the heme iron if it maintains an open axial coordination site. In O_2_-utilizing proteins this typically leads to instant and reversible inhibition of catalysis or function, for example in CYP and NOS enzymes [77,78] or in the terminal respiratory oxidase [79,80]. In hemeproteins that do not utilize O_2_ such as sGC or the nitrophorins, heme-NO binding can activate their functioning in signal transduction cascades [81] or in insect feeding [82], respectively. NO can also impact hemeproteins by it ultimately causing protein modifications like S-nitrosation of protein Cys residues (SNO) or Tyr nitration. These modifications typically diminish hemeprotein functions and the bound heme can sometimes promote these NO-based modifications [83,84]. A third way that NO can impact hemeproteins is by regulating their heme allocation in cells, which can lead to both positive and negative outcomes in their heme contents [85]. Overall, these various impacts of NO can combine to cause complex effects on hemeprotein functions in biology.

### 1.14. Low NO exposures cause cells to allocate heme to apo-hemeproteins to generate their function forms

Exposing cells to low NO concentrations, or in some cases to high NO concentrations for a short duration, have been found to stimulate cell heme allocation into the apo-hemeprotein populations. This was first indicated based on an 10 min NO exposure causing the apo-sGC-β subpopulation in cells to change its protein partners (lose Hsp90 and gain sGC-α) and change its drug response profile in a manner that indicated it had likely gained heme content [24]. This was later substantiated using a sGC reporter protein (TC-sGC-β) whose fluorescence emission after being FlAsH-tagged is inversely related to its heme content [25]. The use of TC-sGC-β also allowed measurements in real time that revealed NO exposure triggered cells to begin allocating heme into apo-TC-sGC-β within seconds [25], indicating that NO exposure mobilized a readily-available existing heme store. NO-driven heme incorporation into apo-sGC-β required participation by cell GAPDH and Hsp90 just like during its normal maturation and caused the sGC-β subunit to lose Hsp90 and bind with a sGC-α subunit to form a functional heterodimer. Cell heme allocation to apo-sGC-β could be driven by very low levels of NO, as demonstrated by it being triggered by the NO released into culture by neighboring cells expressing nNOS [25]. Subsequent studies revealed that low NO concentrations stimulated cell heme allocations to several different hemeproteins including Hb-α, − β, and -γ, myoglobin, NOS, myeloperoxidase, NOX5, IDO1, TDO, CYP3A4, and CYP2D6 [26,86,87] (Sinha et al. 2025 in review). In all cases the NO-driven increase in their heme contents was nearly complete and resulted in a doubling or tripling of the heme-containing subpopulation in the cells along with proportional increases in their function (i.e., catalytic activities in the case of enzymes).

A study with purified proteins to investigate the mechanism of NO action showed that it increased the rate of heme transfer from a GAPDH heme complex into apo-sGC-β by two-fold, through NO binding to the heme iron and causing the rate of heme dissociation from GAPDH to increase by two to three-fold [53]. This NO effect on GAPDH heme affinity may help explain how NO exposure stimulates GAPDH heme allocation to apo-hemeproteins in cells. Overall, low NO exposure is emerging as an environmental signal that causes cells to allocate heme into a wide range of hemeproteins via GAPDH- and Hsp90-dependent mechanisms, thus increasing the level of functional hemeproteins independent of any gene activation.

How low NO concentrations may impact mitochondrial heme allocation to and from GAPDH in cells was recently investigated. Using TC-GAPDH, it was shown that low NO exposure neither caused cells to increase or decrease the level of heme bound in GAPDH [49]. However, in cells that were also expressing a client hemeprotein like IDO1, the low NO exposure did cause TC-GAPDH to transfer its existing heme to the apo-IDO1 population. A NO-triggered redistribution of heme from the cell GAPDH-heme pool to apo-IDO1 would be consistent with its ability to lower the heme affinity of GAPDH in the defined reconstitution system [49]. Together, this implies that low NO concentrations shift the heme binding equilibrium between the GAPDH heme pool and the client apo-hemeprotein in cells to favor the apo-hemeprotein. Thus, low NO exposures can dynamically increase the pool of mature and functional hemeproteins in cells through its effect on this heme binding equilibrium.

### 1.15. High or chronic NO exposure often inhibits hemeprotein function

Negative impacts of NO on hemeproteins have typically been observed in systems due to the common use of relatively high or long NO exposures. Early studies showed that relatively high NO exposure for 12 h severely diminished the heme contents of a broad range of hemeproteins expressed in mammalian cells [40,88]. Animal models of inflammation have revealed that increases in NO production could likely explain why the inflammation diminished hemeprotein activities, particularly for CYP drug metabolism in experimental animals or as seen in patients [89]. In a few cases NO was suggested to inhibit by causing heme loss [77]. Heme loss was also imagined to explain how NO inactivates sGC but recent work showed it does not primarily involve heme loss [67] [71,90]. Instead, alternative mechanisms such as NO-promoted buildup of protein SNO residues may mediate inactivation of both sGC and NOS, for example by destabilizing their functional dimeric structures [71,90–93].

Higher NO exposure can also negatively impact hemeproteins through it having a negative effect on cell heme allocation. This was first shown for iNOS [39], where NO inhibition of its heme allocation in cells was linked to buildup of a SNO modification at a Cys residue in GAPDH (Cys152), and could be rescued by expression of the SNO-resistant GAPDH C152S variant in the cells [41]. This implicated SNO modification in NO downregulation of the GAPDH heme chaperoning function. A subsequent report showed that the SNO-GAPDH buildup in cells exposed to NO was inversely related to the cell’s expression level of thioredoxin reductase 1 (Trx1), an enzyme that can convert SNO-GAPDH back to GAPDH [94]. Indeed, overexpressing Trx1 in the cells rescued iNOS heme allocation from NO inhibition, while knockdown of cell Trx1 expression made it hyper-sensitive toward NO [94]. Thus, in cells undergoing relatively high NO exposure, the SNO modifications that built up in GAPDH negatively impacted iNOS maturation to functional form by inhibiting the cell’s GAPDH-dependent heme allocation. Whether GAPDH-SNO buildup can also explain how higher NO levels limit the heme contents of other GAPDH client hemeproteins like Hb, Mb, CYP, and catalase [40] is an important question to explore.

### 1.16. A bimodal impact of NO on heme allocation

Because NO effects are often determined by the concentration and duration of its exposure it can have both positive and negative impacts. In general, this type of response is called hormetic and is commonly observed in NO biology [95]. Recently the NO-concentration responses for cell heme allocations to the hemeproteins sGC-β, IDO1, and TDO were examined and were found to be practically identical [86,87]. By utilizing a chemical NO donor that releases NO at a precise rate, it was found that the same range of low NO donor concentrations increasingly stimulated cell heme allocations into the apo-hemeprotein populations of these three hemeproteins, but after an optimal NO donor concentration was reached, further increasing its concentration resulted in a gradual loss of the positive effect and ultimately caused some heme loss from the TDO and IDO1 hemeproteins [86,87]. The positive effect of low NO exposure began immediately and took about 4–6 h for the heme contents of the hemeproteins to achieve new equilibrium values, with exposure to the optimal NO donor concentration resulting in full heme saturation. In contrast, only a steady heme loss from the TDO and IDO1 hemeproteins was observed in the cells being exposed to the highest NO concentrations [87]. In all cases, the positive or negative changes in the hemeprotein heme contents tracked with parallel changes in their enzymatic activities.

## 2. NO effects on cell heme allocation are dynamic and reversible

Cells expressing TC-GAPDH along with IDO1 were utilized to investigate how consecutive exposure to a low then high NO donor concentration would impact the distribution of the existing heme between TC-GAPDH and IDO1 [49]. As expected, the low NO donor exposure caused the existing heme in TC-GAPDH to transfer into the apo-IDO1 population. Remarkably, the subsequent higher NO donor exposure caused the heme in IDO1 to transfer back to the TC-GAPDH, indicating it had caused the process to reverse. This revealed that NO effects on cell heme distribution can be dynamically reversible and can go in either direction depending on the NO concentration. The mechanisms by which NO caused the forward or reverse heme exchanges between GAPDH and the IDO1 hemeprotein are unlikely to be the same, and studies uncovering the mechanisms and the scope of this behavior among hemeproteins are awaited with interest.

### 2.1. A model linking the hormetic effect of NO on hemeprotein heme content, function, and biology of the organism

**Fig. 4.**
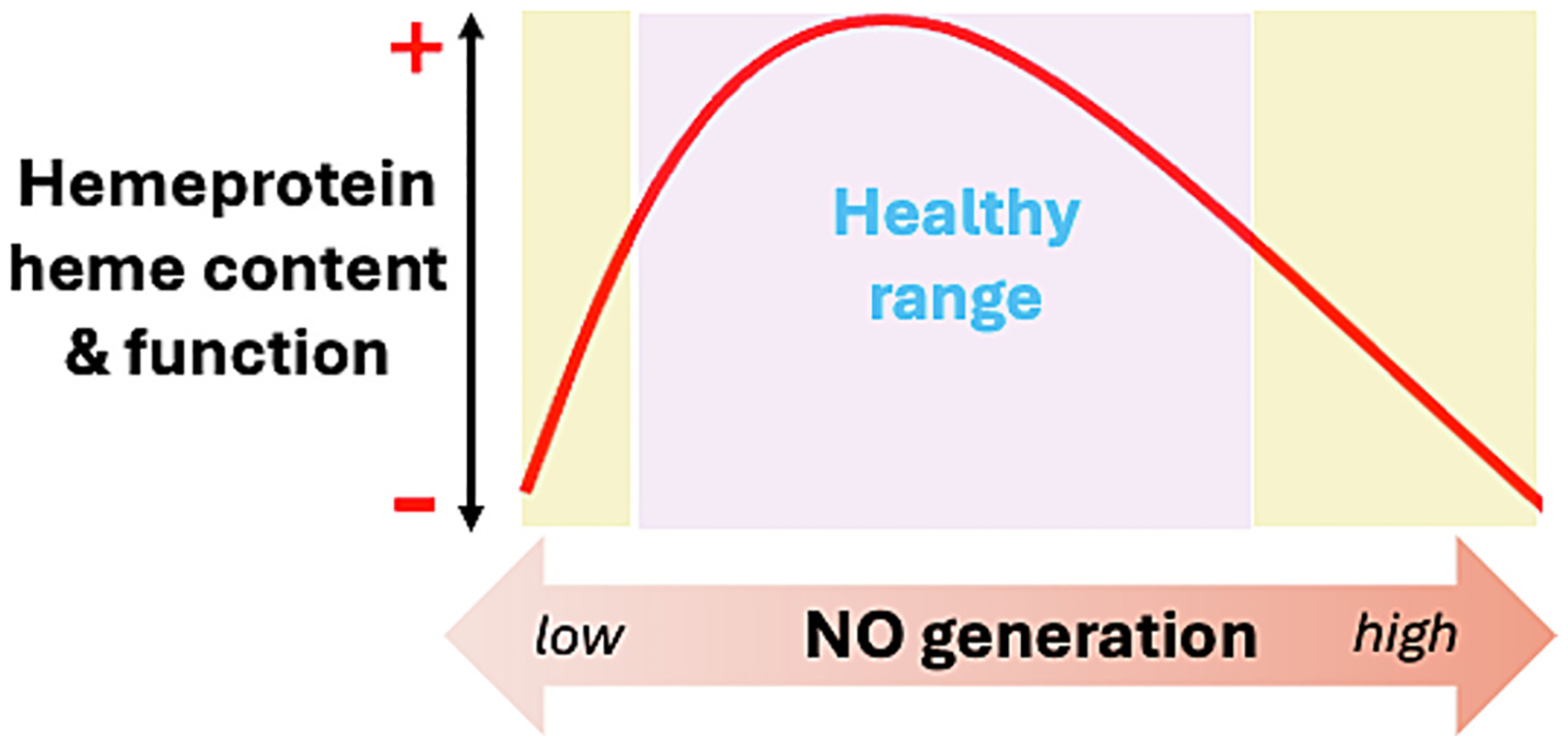
Impact of NO on the heme content and function of hemeproteins and its possible relationship to health and disease. Gradual increases in cell or tissue NO generation can cause graded increases in the heme contents and functions of hemeproteins, but after an optimal level of NO generation is reached, further increases lose their positive effect and at the highest levels can cause loss of heme and hemeprotein function. Perhaps in health a range of NO generation levels is maintained (purple background) that beneficially regulate the heme levels and functions of hemeproteins, while NO generation levels below or beyond this range (yellow background) may cause suboptimal heme levels and functions in hemeproteins that lead to disease.

Mammals naturally express three NO-generating NOS enzymes in their tissues [96] and can also convert dietary nitrate into NO [97]. Measurements show that healthy humans generate a range of NO levels that can diverge further with age, inflammation, or disease [98–100]. Perhaps in health, humans generate a tonic range of NO concentrations to support a level of heme allocation that regulates their hemeprotein heme contents and functions to levels that are biologically near-optimal, all things considered (Fig. 4). Deviations below or above this tonic range, as may occur in aging or disease, may negatively impact hemeprotein functions by supporting less than optimal heme allocation due to lower or higher NO (Fig. 4). In addition, the point where the level of NO generation transitions from being beneficial to negative for hemeprotein functions could be influenced by various circumstances, for example those that modulate NO lifetime in biological settings (super-oxide generation) or that can govern the buildup of impactful NO-derived protein modifications like GAPDH-SNO formation. In general, this description of how the cell’s hormetic response to NO can positively or negatively influence heme allocation may help to explain the multiple and sometimes contradictory impacts that NO has on hemeprotein functions and may help to uncover the true potential of NO in regulating hemeproteins in biology.

## 3. Conclusions & perspectives

Due to heme’s promiscuous binding and inherent reactivity, its transport in animal cells presents a considerable challenge. Recent work has uncovered new insights, including: (i) Non-erythroid cells and tissues maintain a level of exchangeable heme that is insufficient to saturate their hemeproteins, thus making heme allocation an additional way for cells to regulate the functions of a large set of hemeproteins. (ii) Most apo-hemeproteins exist in complex with Hsp90, which governs their protein partner interactions and enables their heme insertions in an ATP-dependent manner. (iii) The GAPDH-heme complex is an essential intermediate species in cells for heme allocation. It associates with the apo-hemeproteins and may directly deliver its heme. (iv) Multiple environmental signals can dynamically influence heme distribution in cells and in their hemeprotein populations. This includes NO, which fundamentally impacts cell heme allocation and causes positive or negative changes in the heme levels of hemeproteins.

These concepts prompt some interesting questions. For example, given the capacity of mammalian cells to generate heme, why do they limit their hemeprotein functions by maintaining them in heme deficient states? Four possible explanations come to mind: (1) It provides a way for cells to regulate their heme protein functions independent of changes in their gene expression. (2) It allows heme to serve as a signaling molecule within cells [101]. Indeed, the functions of heme-responsive transcription factors and kinases are proportional to their bound heme contents, and keeping these proteins sub-saturated allows them to respond to changes in cellular heme levels. (3) It reflects a practical compromise to balance the beneficial effects of heme versus the toxic effects that arise when it is in excess [102]. The heme iron is redox active and can catalyze generation of reactive oxygen species, so perhaps on balance keeping heme levels below some threshold is preferable to a fuller saturation of cell hemeproteins. (4) It reflects a survival adaption that limits the ability of non-erythroid cells to be an iron source for invasive microbes [103] [104,105]. These and other possibilities can now be examined.

A related question regards an apparent paradox in intracellular heme trafficking: Based on thermodynamics and the reported high heme affinities of many hemeproteins (Hb, CYP450, NOS, etc.), shouldn’t they be more fully heme-saturated in the cells? The simple explanation is that heme distribution in cells is not primarily determined by the thermodynamics of heme binding in these proteins. As reviewed here, the way that heme travels from the mitochondria to apo-hemeprotein targets in cells is not random or direct but instead primarily goes through a specific intermediate carrier protein, GAPDH, whose association with the mitochondrial heme exporter FLVCR1b allows it to obtain heme, and whose subsequent association with client apo-hemeproteins (or possibly in some cases via middlemen proteins) enables them to receive heme. Restricting heme transfer out of the mitochondria to a specific protein that in turn must engage in specific protein associations at the molecular level with downstream intermediaries or with the clients themselves creates barriers that disfavor a more random leaky heme distribution based simply on thermodynamic considerations. In addition, Hsp90 binding to the apo-hemeproteins likely helps to determine their observed heme saturation levels. This is because the apo-hemeprotein-Hsp90 complex is the actual recipient of intracellular heme delivery, and so its heme affinity becomes primarily important. As noted previously, Hsp90 likely holds apo-hemeproteins in conformations that make their heme sites more accessible for heme entry. This circumstance would also increase the rate of heme dissociation from these sites, and thus lower the overall heme affinity of the apo-hemeproteins when in complex with Hsp90 compared to the affinities that have been measured for their fully-folded heme containing forms, which primarily are based on measured heme dissociation rates. Indeed, in the context of intracellular heme trafficking, the heme affinities measured for fully folded, heme-replete hemeproteins are probably not so useful for predicting the heme distribution landscape in cells. Rather, the evidence suggests that for heme distribution a near equilibrium is reached between a pool of GAPDH-bound heme and the apo-hemeprotein client, which is typically in complex with Hsp90, and this equilibrium is privy to the associations made by GAPDH to acquire heme, to the inherent heme affinity of the Hsp90-apo-hemeprotein complex, and to the ATP-driven conformational changes in the Hsp90-apo-hemeprotein during the heme transfer. Only after Hsp90 dissociates and the fully folded heme-containing hemeprotein is generated can its heme affinity impact the overall distribution equilibrium. Together, this specific multistep heme transfer creates kinetic barriers that places the thermodynamics of heme binding in the fully folded hemeprotein at a considerable distance from the heme distribution equilibrium between GAPDH and the Hsp90-apo-hemeprotein complex. Indeed, the finding that NO may increase the heme content of hemeproteins in cells by it binding to the GAPDH-heme complex to increase the rate of heme dissociation suggests that the heme distribution equilibrium between GAPDH and the Hsp90-apo-hemeprotein complex is preeminent for determining the overall heme distribution outcome. In future it will be interesting to determine how widespread is partial heme saturation among different cell hemeproteins, and why a hierarchy in their heme saturation levels seems to exist among different organ types. Studies that further elucidate the molecular mechanisms of GAPDH, Hsp90, and NO in intracellular heme deliveries, and that explore if defects in their behaviors causes aberrant hemeprotein function that underpin disease, are also awaited with interest.

## Data Availability

No data was used for the research described in the article.
